# Caveolin-1: an ambiguous partner in cell signalling and cancer

**DOI:** 10.1111/j.1582-4934.2008.00331.x

**Published:** 2008-04-08

**Authors:** Andrew F G Quest, Jorge L Gutierrez-Pajares, Vicente A Torres

**Affiliations:** FONDAP Centre for Molecular Studies of the Cell, Facultad de Medicina, Universidad de Chile

**Keywords:** caveolin-1, cancer, metastasis, multi-drug resistance, apoptosis, proliferation

## Abstract

Caveolae are small plasma membrane invaginations that have been implicated in a variety of functions including transcytosis, potocytosis and cholesterol transport and signal transduction. The major protein component of this compartment is a family of proteins called caveolins. Experimental data obtained in knockout mice have provided unequivocal evidence for a requirement of caveolins to generate morphologically detectable caveolae structures. However, expression of caveolins is not sufficient *per seto* assure the presence of these structures. With respect to other roles attributed to caveolins in the regulation of cellular function, insights are even less clear. Here we will consider, more specifically, the data concerning the ambiguous roles ascribed to caveolin-1 in signal transduction and cancer. In particular, evidence indicating that caveolin-1 function is cell context dependent will be discussed.

- Introduction
- The caveolins
- Caveolin-1 in cell physiology
  - Caveolin-1 distribution
  - Caveolin-1 and internalization
  - Caveolin-1 and cholesterol
  - Regulation of caveolin-1 expression
  - Caveolin-1 in signal transduction
  - Alternative mechanisms of caveolin-1-mediated control in signalling
    - Control of transcription
    - Other modes of control
  - Cell proliferation
  - Cell death and apoptosis
- Caveolin-1 in cancer
  - The tumour suppressor hypothesis
  - Caveolin-1 in multi-drug resistance and metastasis
- Concluding remarks

## Introduction

Caveolae were initially discovered by electron microscopy more than 50 years ago and described as flask-shaped invaginations of the plasma membrane [1, 2]. Similar structures are found in a wide variety of differentiated cells, including adipocytes, pneumocytes, fibroblasts, smooth and striated muscle cells, as well as endothelial cells [3]. Caveolae have been considered to be a subgroup within the category of membrane microdomains referred to as lipid rafts that are enriched in lipids including cholesterol and sphingolipids (GM1, sphingomyelin and ceramide). The specific composition results in a higher degree of organization of the lipid constituents in these membrane microdomains, known as the liquid-ordered state, as well as the characteristic property of insolubility in non-ionic detergents at lower temperatures that permitted biochemical characterization of such domains [4, 5]. In the past years, considerable controversy has arisen over what constitutes a lipid raft. However, it is beyond the scope of this review to discuss this aspect in sufficient detail. The interested reader may wish to consider additional references discussing related issues [6–8].

Unlike lipid rafts, which represent planar, morphologically indistinguishable regions of the plasma membrane, caveolae are coated by a unique family of proteins, termed caveolins that oligomerize to generate large macromolecular complexes. In doing so, caveolins are thought to define caveolae architecture and morphology perceived by microscopy [9–12]. Consistent with this view, caveolin concentration is high in caveolae (100–200 molecules per caveolae) [13] and they have been proposed to represent the structural proteins required for membrane bending that generates the typical 60–80 nm invaginations of the plasma membrane (see discussion in [14]). Alternatively, caveolae formation may require association of other proteins that are then stabilized in the presence of caveolins [15]. Here, it is important to emphasize the fact that caveolae morphology is not invariant. Depending on the cell type and the circumstances, caveolae also can be flattened out within the plane of the membrane or accumulate either individually or as multivesicular clusters beneath the plasma membrane [14, 16].

## The caveolins

The caveolin family is composed of the three isoforms in mammalian cells, caveolin-1, -2 and -3 [17–19]. Caveolin-1 is expressed in a variety of tissues. Caveolin-3, however, is more restricted in its distribution and has been described particularly in muscle [20] and also, more recently, in glial cells [21]. Caveolin-2 is usually co-expressed with caveolin-1, but its function is less well understood. Caveolin-1 forms homo-dimers, as well as heterodimers with caveolin-2. These aggregate into oligomeric structures containing 14–16 molecules in the Golgi and then coalesce into large macromolecular complexes that define caveolae architecture at the plasma membrane or possibly before hand in the Golgi (see discussion in [14]). In caveolin-1 knockout mice, caveolae are only detectable in skeletal and heart muscle cells [9, 10]. In knockout mice for caveolin-2, no changes in caveolae number or morphology are detectable [22, 23]. Caveolin-3 knockout mice lack caveolae in skeletal and heart muscle [24]. Double knockout mice for caveolin-1 and -3 are completely devoid of caveolae [12]. Together with a large body of data available in the literature, these results corroborate the notion that either caveolin-1 or caveolin-3 is required to form caveolae. Caveolin-2 appears less important in this respect, although presence of this isoform does modulate efficiency of caveolae formation, as well as morphological traits of caveolae. Also other proteins appear important in this respect (see discussion in [14, 25]). In particular, the cytosolic protein PTRF-cavin, initially identified as a transcription factor, was recently shown to represent an abundant caveolar coat protein that is required for stabilization of the characteristic flask-shaped plasma membrane invaginations and probably mediates interactions of these structures with the cytoskeleton [26, 27].

Two variants of caveolin-1 have been described, caveolin-1α (residues 1–178) and the 3 kD-smaller caveolin-1β (residues 32–178), which is either generated from full-length mRNA by alternative initiation or by transcription of a shorter splice variant [28–30]. Interestingly, the shorter caveolin-1β variant is mainly present in shallow caveolae and at the leading edge of migrating cells (see Table 1, [31]). Coincident with the possibility that caveolin-1α and -1β may fulfill distinct functions, different roles have been ascribed to the two in zebrafish development [32].

**Table 1:** Caveolin-1 localization in cells using different techniques and antibodies

Caveolin-1 isoform	Post-translational modification	Subcellular localization	Type of cell	Antibody	Technique	Reference
Cav-1 α		Deep caveolae	Human skin fibroblast	Rabbit polyclonal sc-894 (Sta Cruz), mouse monoclonal clone 2234 (Transduction Lab)	Freeze-fracture immunoelectron microscopy	[31]
Cav-1 α/β		Deep and shallow caveolae		Mouse monoclonal clone Z034 (Zymed Lab), mouse monoclonal clone 2297 (Transduction Lab)		
Cav-1 α		Micropatches within the cell, less prevalent along the edge of the cell	FRT (rat thyroid epithelial cell line)	Mouse monoclonal (Transduction Lab clone 2234)	Confocal immunofluorescence	[30]
Cav-1 α/β		Intense accumulation of micropatches along the leading edge	FRT (rat thyroid epithelial cell line)	Mouse monoclonal (Transduction Lab clone 2297)		
Cav-1		Rear of planar migrating cell Front of transmigrating cell	Bovine aortic endothelial cell	Rabbit polyclonal (BD Biosciences)	Immunofluorescence microscopy	[209]
GFP-Cav-1		Cell-cell contact sites	NIH3T3 (mouse fibroblast cell line)		*In vivo* fluorescence microscopy	[116]
Cav-1		Focal adhesions	Senescent human foreskin fibroblasts	Mouse monoclonal (C43420, Transduction Lab)	Immunofluorescence confocal microscopy	[210]
Cav-1		Cytoplasm	Human keratinocytes and fibroblasts		Immunofluorescence	[65]
Cav-1		Nucleus (nuclear matrix and chromatin)	SKOV3 (human ovarian carcinoma cells)	Mouse monoclonal (Santa Cruz)	Immunofluorescence confocal microscopy, chromatin immune precipitation, GFP-Cav-1	[72]
Cav-1		Secretory vesicles	Mouse salivary gland, anterior pituitary		Immunofluorescence and immunogold detection	[65]
Cav-1 α/β		Mitochondria	Rat liver		Immunoblotting, immunoprecipitacion, electron microscopy immunogold detection	[65]
Cav-1	pY14	Cytoplasmic vesicles when treated with pervanadate or hydrogen peroxide	Human umbilical vein endothelial cells and bovine aortic endothelial cells	Rat monoclonal	Immunofluorescence microscopy	[211]
Cav-1	pY14	Focal adhesions	NIH3T3 (mouse fibroblast cell line)	Mouse monoclonal (Transduction Lab clone 56)	Immunofluorescence microscopy	[46]
Cav-1	pY14	Focal adhesions	NIH3T3 expressing v-Src	Mouse monoclonal (Transduction Lab clone 56)	Immunofluorescence confocal microscopy	[39]
Cav-1	pY14	Cytoplasmic and flat intramembrane particle-free area	src^ts^ NRK (rat kidney cell line expressing temperature sensitive src)	Rabbit antiserum	Immunofluorescence microscopy and freeze fracture immunoelectron microscopy	[212]

Caveolin-1 was initially described as a prevalent target for tyrosine phosphorylation in Rous sarcoma virus transformed chicken fibroblasts [33–35]. Importantly, for the subsequent discussion, only caveolin-1α contains a tyrosine residue at position 14 that is frequently phosphorylated in response to cell stimulation by agonists including insulin [36–38], epidermal growth factor (EGF) [39, 40], platelet-derived growth factor (PDGF) in conjunction with loss of sterols [41], a progesterone analogue [42], mechanical stress [43] and oxidative stress [44–46]. Interestingly, however, whether phosphorylation occurs at this site in response to a stimulus is cell dependent. In adipocytes, for instance, tyrosine phosphorylation of caveolin-1 is observed only in response to insulin, but not with EGF PDGF, tumour necrosis factor (TNF)-α or interleukin (IL)-6 [39]. Non-receptor src-family tyrosine kinases, such as c-Src and c-Abl, have frequently, but not exclusively, been implicated in caveolin-1 phosphorylation at this site (see [36–46]).

In addition to caveolins-1α and -1β, three different isoforms of caveolin-2 (caveolin-2α, -2β and -2γ) have been detected, although these are less well characterized [23, 47]. Caveolins-1 and -2 are broadly expressed, while caveolin-3 expression is limited to muscle and glial cells [20, 21]. Genes encoding each family member share significant homology. Also caveolin homologs have been described in zebrafish *D. rerio*[32] and the nematode *C. elegans*[48].

The caveolin-1 sequence contains a hydrophobic central domain between residues 102 and 134 that is inserted in the inner leaflet of the plasma membrane. This region adopts a hairpin-like conformation, thereby exposing both COOH- and NH_2_-terminus to the cytoplasm (see [14, 16]). In the NH_2_-terminal region, immediately adjacent to the hydrophobic domain (residues 82–101), a modular sequence, termed the ‘caveolin scaffolding domain’ (CSD) is required for caveolin dimerization, as well as interactions between caveolin-1 and numerous signalling proteins that contain a ‘caveolin binding domain’ (CBD, see next sections). In addition, a WW-like domain initially described in caveolin-3 [49], may also be present in caveolin-1 (residues 98–132; [50]) and play a role in caveolin-1-mediated degradation of the inducible isoform of nitric oxide synthase (iNOS) *via* the proteasome pathway [51, 52]. A combination of elements is required for directing caveolin-1 to the Golgi (amino acids 66–70), oligomerization (amino acids 91–100 and 135–140) and transport to the cell surface (amino acids 71–80) [53, 54]. The COOH-terminal region contains three palmitoylated cysteine residues that are not required for caveolin-1 transport or localization to caveolae, but are relevant to oligomerization [55, 56], as well as two separate regions implicated in cross-linking caveolin dimers [57]. Furthermore, a minimal sequence of 10 amino acids (46–55) was defined in the amino terminal region of caveolin-1 that is required for localization of the protein to the rear of migrating cells and formation of caveolae [58].

In summary, caveolins are evolutionary conserved proteins that contain multiple elements relevant to their function distributed throughout the sequence and are crucial components of caveolae. Additionally, overlapping (scaffolding domain) or unique elements (tyrosine 14) are utilized in signal transduction. Given that the majority of data available in the literature focuses on caveolin-1, we will centre the rest of the discussion on that isoform.

## Caveolin-1 in cell physiology

**Fig. 1:**
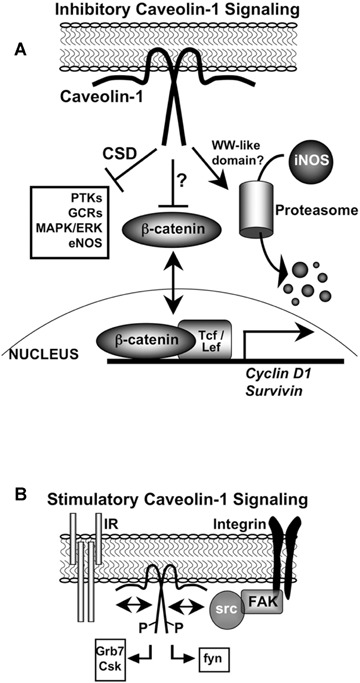
Caveolin-1 is a highly versatile regulator of cell signaling. (**A**) At the molecular level, Caveolin-1 can modulate the flow of information through different cellular signaling pathways in distinct ways. Caveolin-1 acts as a scaffolding protein by binding to proteins involved in different signal transduction pathways. The caveolin-1 scaffolding domain (CSD) mediates interaction with and inhibition of protein tyrosine kinases (PTKs), G-coupled receptors (GCRs), elements of the MAPK/ERK pathway, members of the protein kinase C (PKC) family and nitric oxide synthases (NOS), in particular the endothelial isoform (eNOS). Additional mechanisms to CSD-mediated inhibition of target proteins that result in loss of target protein activity, include proteasome-mediated degradation as has been described for the inducible isoform of nitric oxide synthase (iNOS). Furthermore, inhibition of β-catenin-Tcf/Lef-dependent transcription of genes such as cyclin D1 and surviving by poorly characterized pathways has been observed. The general consequence of these interactions is that caveolin-1 presence is associated with inhibition of cell proliferation and/or survival. (**B**) Alternatively, however, caveolin-1 has been implicated as a positive element in insulin receptor (IR) signalling and coupling of the integrin signalling to the MAPK/ERK pathway *via* focal adhesion kinase (FAK) and src family kinases (SFKs). Such positive signalling downstream of caveolin-1 is frequently associated with phosphorylation on tyrosine 14, whereby downstream positive effectors include Csk, Grb7. Alternatively, however, positive signalling *via* fyn downstream of integrin receptors is not known to require caveolin-1 phosphorylation. Rather, phosphorylation on tyrosine 14 is implicated as a negative regulator of rac-1 in conjunction with integrin signalling (see text for details).

**Fig. 2:**
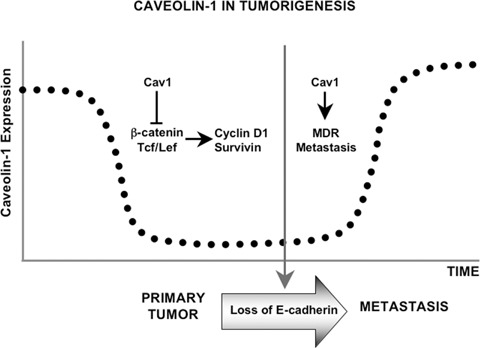
Caveolin-1 in tumorigenesis. The dual nature of caveolin-1 in cell signalling is reflected at the functional level by data indicating that caveolin-1 develops either tumor suppressor or tumor promoting activities depending on the tumor type under study. In breast, lung, colon and gastric cancer, initial loss of caveolin-1 is thought to promote cell proliferation and reduce apoptosis. In colon and other cancers, including melanomas, such effects may be associated with enhanced β-catenin-dependent transcription. However, during tumor progression a number of additional changes occur at the molecular level that not only reduce the ability of caveolin-1 to function as a tumor suppressor, but then generate a “permissive” cellular environment that allows caveolin-1 to operate in the opposite fashion. Here loss of E-cadherin is mentioned as one possibility. This model serves to explain how expression of caveolin-1 at later stages of tumor development may be associated with higher metastatic potential, multi-drug resistance (MDR) and poor patient survival. In this respect, the model is also applicable to cases like prostate cancer where caveolin-1 is not expressed in the normal tissue. In summary, the model proposed implicates caveolin-1 as a conditional tumor suppressor that operates in a cell context-dependent fashion.

Given the number of caveolin-1 elements implicated in mediating protein-protein and protein-lipid interactions, it is not surprising that the protein is detected at many locations throughout the cell and implicated in a wide variety of processes, including vesicular transport (endocytosis, transcytosis and potocytosis), cholesterol homeostasis and regulation of signal transduction [3, 18, 53, 59, 60]. Particularly in the latter case, the data available are confusing. On the one hand, caveolin-1 blocks many signalling events *via* interactions requiring the scaffolding domain (Fig. 1A and references above). On the other hand, the protein is implicated as a positive regulator in, for instance, integrin, insulin and progesterone signalling (see Fig. 1B; [42, 59, 61, 62]). This ambiguous relationship combined with variations in subcellular distribution, provide a potential rationale to understanding how caveolin-1 presence in tumour cells may in some cases be associated with tumour suppression, but in others, with more malignant phenotypes including multi-drug resistance and metastasis (Fig. 2). The following paragraphs will focus on the discussion of such issues.

### Caveolin-1 distribution

Caveolin-1 is an integral membrane protein that is generally found associated with membranous structures, including the endoplasmic reticulum (ER), Golgi and plasma membrane, as might be expected since the elements responsible for transit from ER to the plasma membrane *via* Golgi have been defined. When synthesized, caveolin-1 is co-translationally inserted into the ER, facing the cytoplasm with both its NH2- and COOH-terminal sequences. Thereafter, caveolin-1 is shipped to the Golgi where it oligomerizes and is concentrated within cholesterol- and sphingolipid-rich membrane microdomains. Finally, caveolin-1 oligomers are transported to the cell surface to form caveolae. From the plasma membrane, caveolae can recycle back to intracellular compartments including the ER and the caveosome [54, 60, 63].

Caveolin-1 is found in additional locations within the cell that are often difficult to understand within the framework of the scheme outlined above. For instance, caveolin-1 is associated with subcellular compartments like mitochondria and is present within the ER lumen and in secretory vesicles, as well as in the cytoplasm as a soluble protein, in focal adhesions, at cell-cell contact sites and even in the nucleus (see Table 1). What determines caveolin-1 presence there remains unclear. This is particularly important given the observation that in some cell types caveolin-1 association with or presence within these structures prevails (see for instance [64, 65]). The potential relevance of these non-conventional pools of caveolin-1 protein is underscored by the fact that secretion of caveolin-1 is associated with enhanced metastatic potential of some tumour cells [66]. Furthermore, a small but significant pool of caveolin-1 is embedded in a lipid particle that is important for cytoplasmic shuttling of cholesterol from the ER to the plasma membrane [67]. Additionally, ER accumulation of caveolin-1 due to over-expression or upon treatment with drugs like Brefeldin A targets caveolin-1 to lipid droplets. However, the role of caveolin-1 in these structures remains controversial [68–71]. Finally, nuclear caveolin-1 has been implicated in transcriptional control [72].

As mentioned previously, caveolin-1 is phosphorylated on tyrosine-14 (caveolin-1(PY14)) in response to a number of stimuli. More recently, considerable controversy has arisen concerning the precise localization of caveolin-1(PY14). While some studies have shown good co-localization between caveolin-1 and caveolin-1(PY14) as might be expected, others indicated that caveolin-1(PY14) accumulated in focal adhesions where caveolin-1 was not detected (see Table 1). This somewhat confusing observation was explained by suggesting that the two antibodies employed in the latter studies bind to overlapping epitopes on the caveolin-1 protein. However, the monoclonal antibody against caveolin-1(PY14) was subsequently shown to also label focal adhesions in cells from caveolin-1 knockout mice. Most recently this controversy was resolved by showing that the monoclonal antibody is specific for caveolin-1(PY14) on western blots, but recognizes phosphorylated focal adhesion proteins, such as paxillin, in immunofluorescence studies (see discussion in [73]).

Additional studies will be required to understand how differences in localization of caveolin-1 are defined. A better understanding of the mechanisms responsible for determining where caveolin-1 is found in a cell and how variations in this distribution pattern impact on cell function is likely to help in defining the contribution of this protein to human disease states including cancer. A specific example that highlights this point involves formation of a caveolin-1/E-cadherin complex at the plasma membrane and is mentioned at the end of this article (see Fig. 2).

### Caveolin-1 and internalization

Caveolae are implicated in internalization *via* mechanisms that are distinct from those described for classic endocytosis [74]. Although several alternatives to clathrin-mediated endocytosis exist where caveolae or caveolae-like domains have been implicated, including potocytosis [75, 76], transcytosis [77, 78] and internalization of microorganisms [79, 80], the precise role of caveolin-1 in these events often remains controversial. For instance, caveolin-1 has been proposed to act in internalization in a manner akin to clathrin that recruits receptor molecules into coated pits and also drives membrane invagination in classic endocytosis. Consistent with this type of analogy, caveolin-1 participates in the recruitment of src to cholesterol-rich microdomains required for dynamin-2 phosphorylation, activation and severing of caveolae from the plasma membrane [81–83]. However, more recent evidence indicates that caveolin-1(PY14), rather than the non-phosphorylated protein, is responsible for internalization of membrane microdomains and associated proteins downstream of integrins [84]. Likewise, the EGF receptor is internalized *via* caveolae to a perinuclear compartment following exposure to oxidative stress in a manner that requires src-dependent phosphorylation of caveolin-1 on tyrosine 14 [85]. Thus, while similarities exist, caveolin-mediated internalization clearly differs from clathrin-dependent endocytosis. Additional aspects highlighting these differences are listed below. First, caveolin-1 interacts with many proteins at the plasma membrane via the CSD; however, this interaction is not necessarily what drives presence in the caveolae compartment, as is the case for the EGF receptor, the endothelial isoform of nitric oxide synthase (eNOS), src-family tyrosine kinases, Gαq and the insulin receptor [18, 86, 87]. Second, unlike clathrin-coated endosomes, caveolae are essentially stable vesicular transporters that do not intermix [88]. Third, internalization of cholera toxin (CT) that binds to the raft marker glycosphingolipid GM1 is sensitive to cholesterol depletion and requires dynamin, as might be expected for a caveolae-mediated internalization process. However, CT is also internalized in a similar fashion in caveolin-1 and caveolae-deficient Jurkat and CaCo2 cells. Fourth, not only is caveolin-1 dispensable for CT internalization *via* rafts, but it appears that internalization is slower in the presence of caveolin-1 [15, 47]. Likewise, experiments investigating internalization of SV40 showed that this occurs *via* association with caveolin-1-coated structures and subsequent transport to a novel intracellular, Golgi-proximal compartment called the caveosome [63, 89]. Interestingly, however, endocytosis of SV40 is also observed in cells lacking caveolae *via* clathrin- and caveolin-1-independent endocytosis [90, 91]. Finally, FRAP analysis of GFP-caveolin-1 chimeras reveals that caveolin-1 is present within the cell on highly mobile vesicles, but once inserted into the membrane, the mobility drops dramatically, perhaps due to association with the cytoskeleton [47, 92–94]. Consistent with this view, mobility of plasma membrane-associated SV40 particles drops dramatically upon co-localization with caveolin-1. In the presence of Latrunculin A, an actin monomer sequestering drug that reduces SV40 internalization, such immobilization is not observed [89]. For a more detailed discussion of the regulation of raft-dependent endocytic pathways and the role of caveolin-1 in these events, the interested reader is referred to a recent review [95].

### Caveolin-1 and cholesterol

Early on, following the discovery of caveolins, the relevance of this protein in modulating cholesterol distribution in cells was established. Caveolin-1 was shown to bind directly to cholesterol *in vitro*[96] and loss or absence of caveolin-1 reduces cholesterol transport to the plasma membrane, as well as presence of cholesterol in caveolae or rafts [97]. Also caveolin-1 is implicated in cholesterol accumulation in lipid droplets of adipocytes [69]. More recently, models describing the potential role of aromatic residues of the hydrophobic motif, as well as phosphorylation at S80 in this association have become available [14]. In this context, it is perhaps worth noting that phosphorylation at this same site appears important for regulation of caveolin-1 secretion [98] and, hence, might provide a link to the secreted pool of caveolin-1 associated with metastatic potential of some cancer cells [66].

Given the importance of cholesterol in membrane microdomain formation, extraction or sequestration using reagents like β-methyl-cyclodextrin, filipin or nystatin, as well as cholesterol oxidation, leads to disruption of rafts and caveolae alike and blocks cellular processes, including a plethora of signal transduction events associated with the integrity of such domains [53, 80]. The relevance of these processes to complex cell behaviour, including cell transformation, is underscored by genetic evidence showing that a caveolin-3 mutant that blocks cholesterol transport precludes H-ras-dependent cell transformation [99, 100].

Although disruption of microdomains by cholesterol sequestration generally blocks signalling *via* growth factor receptors (PDGF insulin), such treatments have also been found to promote signalling, for instance, downstream of the EGF receptor in cells expressing caveolins (reviewed in [101]). In T-cells, which typically lack caveolins, lipid rafts are considered hotspots for both lipid-and protein-mediated signalling pathways. Interestingly, within the first few minutes of treatment with β-methylcyclodextrin, transient activation of several signalling pathways is observed. This is then followed by inactivation. These results in T cells can be taken to indicate that cholesterol removal initially promotes mixing of raft and non-raft components that are segregated to maintain the inactive state. Excessive cholesterol depletion leads then to randomization of membrane components, inability to generate the immunological synapse and down-regulation of signalling (see discussion in [102, 103]). Taken together, these data underscore the importance of cholesterol-containing membrane microdomains as structures that are required to help molecules coalesce in a meaningful manner and thereby promote signalling (see discussion in [104, 105]). However the relationship is complex and needs to be assessed on a case-by-case basis. A more detailed discussion of this topic is available elsewhere [80, 101].

### Regulation of caveolin-1 expression

Analysis of patients identified a P132L mutation in caveolin-1 in about 16% of breast cancer samples analyzed [106]. Interestingly, this mutation results in retention of the mutated protein in the Golgi and subsequent degradation. Moreover, the mutant protein acts as a dominant negative that precludes appropriate sorting of endogenous wild type caveolin-1 to the cell surface and also promotes degradation of the wild-type protein. This observation provides an explanation for how mutation of only one allele can result in complete loss of caveolin-1 expression [107]. Interestingly, additional caveolin-1 mutations have recently been found associated with oestrogen-receptor α-positive breast cancers [108]. Despite such data and the fact that post-transcriptional mechanisms of caveolin-1 regulation exist [109], the majority of evidence available suggests that caveolin-1 is an example of a non-classical tumour suppressor protein whose expression is modulated by transcriptional silencing *via* epigenetic changes rather than by mutation [110–114]. The following paragraphs are dedicated to the discussion of mechanisms implicated in the control of caveolin-1 transcription (see Table 2).

**Table 2:** Regulation of caveolin-1 expression in non-transformed and cancer cells

Non-transformed cells							
Cell	Regulation	Effects	Transcription factor	Implicated signaling pathway	mRNA	Protein	Reference
Human fibroblasts	Increase in Cav1	Senescence	Sp1	Oxidative stress/p38	+†	+	[147]
Human skin fibroblasts	Increase in Cav1	Cholesterol efflux	p53		+†	+	[124]
Human lung fibroblasts	Increase in Cav1			PKCα	n.d.	+	[213]
Human macrophage cell line (THP-1)	Increase in Cav1		PPARγ		+*	+	[214]
Human endothelial cell line (ECV304)	Increase in Cav1		Sp1	Cholesterol/SREBP	+†	+	[130]
Human endothelial cells	Increase in Cav1			Shear stress/intracellular calcium	n.d.	+	[215]
Mouse epithelial lung cell line (E10)	Increase in Cav1		Ets		+*^, †^	n.d.	[216]
Mouse peritoneal macrophage	Increase in Cav1			Lipopolysaccharide/p38 (MEK-independent)	n.d.	+	[217]
Mouse myoblasts (C2C12) and NIH3T3 stably expressing insulin receptor	Increase in Cav1	Quiescence	FOXO	Insulin/PI3kinase/PKB/FOXO	+*	+	[122]
Rat aortic smooth muscle cells	Increase in Cav1		Estrogen receptor (raloxifene, 17β-estradiol)		+*	+	[218]
Primary rat pulmonary artery smooth muscle cells and mouse fibroblasts	Increase in Cav1	Anti-proliferative		Carbon monoxide/p38	n.d.	+	[219]
Human endothelial cells	Decrease in Cav1			TNFα	n.d.	+	[215]
Human endothelial cell line (ECV304)	Decrease in Cav1		KLF11 (repressor)		+†	+	[130]
Human (hTERT-HME1) and mouse (HC11) mammary epithelial cell lines	Decrease in Cav1 and Cav2	Lactogenesis		Prolactin/MEK	+*^,^ †	+	[220]
Human umbilical vein endothelial cell line (ECV 304)	Decrease in Cav1 (Cav2 constant)	Cell proliferation		VEGF/MEK	n.d.	+	[221]
Mouse mammary epithelial cell line (COMMA-1D)	Decrease in Cav1	Cell transformation		PDK1/PKCα/β-catenin/c-Myc	n.d.	+	[120]
Mouse fibroblast cell line (NIH3T3)	Decrease in Cav1	Cell transformation	c-Myc (repressor)		+*^, †^	+	[128]
Mouse fibroblast cell line (NIH3T3)	Decrease in Cav1	Cell transformation		Human papillomavirus E6 oncoprotein/p53 (decrease)	+†	+	[222]
Mouse fibroblast cell line (NIH3T3)	Decrease in Cav1 (Cav2 constant)	Cell transformation		H-Ras(G12V)/p42/44	+*^,^ †	+	[115]
				MAPK			
				PKA			
Mouse fibroblast cell line (NIH3T3)	Decrease in Cav1 (Cav2 constant)	Cell transformation		Neu tyrosine kinase/ERK	+*	+	[223]
				Ceramide/ERK			
				v-Src/ERK			
Primary rat astrocytes	Decrease in Cav1 and Cav2	Associated with senescence		cAMP	+*	+	[224]
				TGFα/PI3kinase (p42/p44 MAPK-independent)			
				Histone deacetylase			

Cancer cells							

Cell	Regulation	Effects	Transcription factor	Implicated signalling pathway	mRNA	Protein	Reference
Human ovarian cancer cells	Increase in Cav1	Growth inhibition	Progesterone receptor		+*	+	[225]
Human colon (HT29) and breast (MCF7) carcinoma cell lines	Increase in Cav1 and Cav2		PPARγ		+*	+	[226]
Human ovarian cancer cell line (OVCAR3)	Increase in Cav1		NF-κB	IL-1β	+*	n.d.	[125]
Human prostate cancer model (CWR22)	Increase in Cav1	Growth promotion		PKCɛ/MEK/c-Myc/Androgen receptor	n.d.	+	[227]
Mouse (C4D)/human (T47D) breast cancer cell lines	Increase in Cav1	Proliferation	Progesterone receptor	PI3kinase MEK	+*, †	+	[42]
Human neuroblastoma cells (SK-N-MC)	Decrease in Cav1 and Cav2		Oestrogen-receptor α (ligand independent)	ERα/MEK//DNA methylation	+*	+	[131]
Human ovarian cancer cell lines	Decrease in Cav1			α folate receptor	+*	+	[228]
Human epidermoid carcinoma cells (A431), prostate cancer cells (DU145), non-small cell lung cancer cells (A549)	Decrease in Cav1	Cell invasion		EGF/EGFR	+†	+	[154]

n.d., not determined.

* Northern blot or RT-PCR.

† Cav-1 promoter reporter assay.

The MAPK/ERK pathway has been extensively characterized as a negative regulator of caveolin-1 expression [115]. In proliferating cells where the MAPK/ERK pathway is active, caveolin-1 levels are low. Alternatively, when cells reach confluency, signalling *via* the MAPK/ERK pathway is diminished and caveolin-1 levels increase [116]. Interestingly, antisense oligo mediated down-regulation of caveolin-1 in NIH3T3 cells leads to hyperactivation of the MAPK/ERK pathway [117]. Likewise, in caveolin-1 knockout mice, cell hyper-proliferation observed in different tissues often appears linked to elevated signalling *via* the MAPK/ERK pathway [59, 118]. Thus, on the one hand the MAPK/ERK pathway regulates caveolin-1 expression and on the other hand, caveolin-1 presence reduces signalling through this pathway. Additional signalling pathways involved in regulation of caveolin-1 transcription include protein kinase A (PKA) and src family kinases [60, 115], phosphatidylinositol-3-kinase (PI-3K, [119]), phosphatidylinositol-3-phosphate dependent kinase 1 (PDK1, [120]) and protein kinase C**a** (PKCα, [120]). Also, testosterone promotes transcription of the caveolin-1 gene *via* an androgen receptor-dependent mechanism [121]. Factors directly implicated in transcription of caveolin-1 include Foxo [122], PPARγ[123], p53 [124], NF-κB [125] and Sp1 [126]. Furthermore, the caveolin-1 promoter contains two SREBP-binding elements sensitive to cholesterol content and degree of oxidation [127]. Indeed, caveolin-1 expression is positively regulated by cholesterol, both by increasing mRNA levels and by stabilizing the protein [126]. Unlike the aforementioned transcription factors, c-myc, KLF11 and oestrogen-receptor α suppress caveolin-1 expression [128–131]. Also, oxysterols reduce caveolin-1 expression *via* a transcriptional mechanism [127].

Thus, on the one hand a variety of signalling mechanisms that result in suppression of caveolin-1 expression, as well as several transcription factors that promote transcription of the caveolin-1 gene have been described. An important question that remains to be resolved is how, if at all, these molecular events relate to suppression of caveolin-1 expression observed early in tumour development or re-expression of caveolin-1 detected at later stages (see Fig. 2). Segregation of the data available into mechanisms reported in ‘non-transformed’*versus*‘cancer’ cells does not yield additional insight in this respect (see Table 2). In addition, the recent finding that regulation of caveolin-1 expression downstream of ras differs dramatically between mouse and human cells [132] may indicate that results obtained using mouse (cell) models need to be interpreted with caution when attempting to understand caveolin-1 regulation in human cancer.

### Caveolin-1 in signal transduction

As alluded to previously, caveolin-1 was first described as a highly phosphorylated protein on tyrosine in Rous sarcoma virus transformed chick fibroblasts and a component of caveolae [33–35, 133]. Thus, from the onset, presence and phosphorylation of caveolin-1 were associated with events involved in cell signalling and transformation.

Since then a great deal of effort has been focused on characterizing the proteins associated with caveolin-1 and caveolae. In this respect, the observation that caveolin-1 is identical to VIP21, a major component of detergent-insoluble, non-coated pits [134, 135] turned out to be particularly helpful. Since then, a plethora of purification schemes have been devised to isolate and characterize caveolae and raft components, a large number of which are based on procedures involving cell solubilization in non-ionic detergents at low temperature and subsequent flotation of the light buoyant, vesicle-associated components in a sucrose gradient. Although prone to many artefacts [6, 16, 50], results from these studies helped to identify a large number of proteins and lipids associated with caveolae, many of which are involved in signal transduction [136]. In doing so, the experimental basis was generated supporting the ‘signalosome’ or ‘caveolae signalling hypothesis’, by which caveolae were suggested to play a central role in orchestrating signal transduction, and caveolin-1 was considered essential both to caveolae structure and function [3, 18, 53, 60]. Insights gained from knockout mice, unequivocally demonstrated the importance of caveolin-1 (and caveolin-3) in caveolae formation [9, 10, 12, 24]. Quite unexpectedly, however, the animals are viable and fertile. Thus, while the structural role of caveolin-1 became immediately apparent from the knockout mouse studies, conclusions with respect to the role of caveolin-1 in signal transduction were less clear.

Perhaps the clearest, generally accepted, exception in this respect is the endothelial isoform of nitric oxide synthase (eNOS). Caveolin-1 presence inhibits eNOS *in vitro* and *in vivo*, most likely *via* direct interaction, and in knockout mice eNOS activity is elevated and associated with vascular defects [9, 10]. Additional examples underscoring the role of caveolin-1 as a regulator of cell signalling do exist. For instance, caveolin-1 knockout mice display a premature lactation phenotype that is attributed to hyperactivation of the Jak2/STAT5a signalling pathway due to reduced Jak2 tyrosine kinase inhibition *via* interaction with caveolin-1 [137]. Moreover, increased activity of the MAPK/ERK signalling pathway is associated with neointimal hyperplasia, cardiac hypertrophy and increased sensitivity to carcinogens, while enhanced proliferation of intestinal crypts cells is linked to alterations in Wnt/p-catenin signalling. A more extensive summary of connections between observed pheno-types and possible mechanisms is provided elsewhere [138]. Thus, despite the relatively mild phenotypes detected, knockout mice do display physiologically significant deficiencies. Presumably, the conjunction of effects contributes to the reduced ability of these mice to respond to specific stress situations and, as a consequence, a significant reduction in lifespan is observed [9, 10, 139, 140].

Following the isolation and characterization of caveolae, it quickly became apparent that caveolin-1 not only co-fractionated (and in some cases co-localized) with many signal transduction proteins, such as EGF receptor, PDGF receptor, insulin receptor, src family kinases, PKCs, H-Ras, Raf kinase, 14–3-3 proteins, ERKs, eNOS, Shc, Grb2, mSOS1 and Nck, to mention a few [60], but also inhibited the catalytic activity of a considerable number, including the EGF receptor, PKCα, eNOS, and src family kinases *via* interaction between the CSD and a CBD located within the target protein. Since the interaction between caveolin-1 and target proteins is often inhibitory and many of the target proteins are important in cell proliferation, caveolin-1 was suggested to represent a negative regulator of proliferation and survival, giving rising to the ‘oncosuppressor hypothesis' [18, 60]. Entirely consistent with this interpretation, oncogene-mediated cell transformation coincides with loss of caveolin-1 expression and re-expression of caveolin-1 in transformed cells is sufficient to revert the transformed phenotype [141, 142]. In addition, targeted down-regulation of caveolin-1 leads to hyper-activation of p42/44 MAP kinase and cell transformation [117]. Furthermore, caveolin-1 inhibits signalling *via* the β-catenin/Tcf-Lef pathway [143, 144], promotes cell cycle arrest *via* a p53/p21^Waf1/Cip1^-dependent mechanism [145] and favours premature cell senescence [146, 147].

### Alternative mechanisms of caveolin-1-mediated control in signalling

The aforementioned view of caveolin-1-mediated control in signalling was based on observations linking binding to subsequent inhibition of target protein function. Most of the initial interactions described occurred *via* the CSD in caveolin-1 and the CBD found in the respective target protein (see text above, [18, 23]). However, a considerable number of additional modes of action exist for caveolin-1: (1) Association between caveolin-1 and target proteins needs neither to involve the scaffolding domain nor be direct. (2) The presence of caveolin-1 quite often promotes signalling events. This is particularly apparent where, for instance, caveolin-1 phosphorylation is involved (see insulin and integrin signalling, Fig. 1B). Also, caveolin/caveolae are implicated in a variety of aspects concerning calcium signalling and handling [148–153]. (3) The ability of caveolin-1 to modulate cholesterol transport and distribution in cells and as a consequence signalling was already mentioned briefly (see section, Caveolin-1 and cholesterol). The remaining discussion in this section will focus on the first two aspects listed here.

#### Control of transcription

Work from a number of independent research groups has implicated caveolin-1 as a negative regulator of β-catenin-Tcf/Lef-dependent transcription [143, 144, 154]. Association between caveolin-1 and β-catenin was initially observed in different cell systems [144]. Furthermore, caveolin-1 expression and intracellular distribution depend on cell-cell adhesion in a manner similar to β-catenin [116, 117]. Although it remains unclear whether the interaction between caveolin-1 and β-catenin is direct or indirect, recruitment of β-catenin to caveolae and/or caveolin-1-containing protein complexes at the cell surface is thought to preclude β-catenin Tcf/Lef-dependent transcription of target genes [143, 144]. The first target gene found to be controlled in this manner was cyclin D1 [155]. Caveolin-1 was suggested to promote cell cycle arrest in G_0_/G_1_ and decrease the number of cells in S phase by decreasing cyclin D1 expression [145, 155]. More recently, the inhibitor of apoptosis (IAP) protein survivin was also shown to be down-regulated by caveolin-1 in a β-catenin-Tcf/Lef-dependent manner [143] that appears to depend on the expression of E-cadherin [156]. Reduction of survivin expression provides an explanation for several alterations caused by the presence of caveolin-1, including a decrease in the number of cells in G_2_/M and an increment in the susceptibility to apoptosis [143].

Alternatively, Hunter *et al*. showed that EGF-induced epithelial to mesenchymal transition (EMT) in A431 human epidermoid carcinoma cells involved down-regulation of caveolin-1 and E-cadherin, and as a consequence enhanced Tcf/Lef-dependent transcription *via* a GSK3p-independent pathway. Importantly, E-cadherin expression was augmented by the presence of caveolin-1 [154, 157]. The expression of E-cadherin is reportedly inhibited by β-catenin dependent mechanisms and by the repressor snail [158–160]. Consistent with the notion that caveolin-1 presence can promote E-cadherin expression, caveolin-1 inhibits β-catenin-mediated transcription as well as the expression of snail [154]. Interestingly, caveolin-1 also precludes EMT by promoting membrane localization of E-cadherin and stabilizing adherens junctions *via* inhibition of src [161].

In this respect, it is worth noting that the regulation of β-catenin-dependent transcription by caveolin-1 is likely to be relevant in human cancers, especially those where β-catenin-dependent transcription is altered, such as colon cancer ([162]; see Fig. 2). Caveolin-1 expression is down-regulated both in colon tumour mucosa and stroma when compared to samples of normal tissue from the same patients. Furthermore, caveolin-1 levels were found to be extremely low in a number of different colon carcinoma cell lines and re-expression of caveolin-1 was sufficient to block tumour formation of colon carcinoma cells in nude mice [50, 163]. Also, the re-expression of caveolin-1 in the breast cancer cell lines MCF7 and ZR75 is sufficient to reduce proliferation and promote apoptosis, possibly by a mechanism involving down-regulation of survivin [143]. Consistent with these observations, the absence of caveolin-1 *in vivo* in knockout mice leads to hyper-proliferation and enhanced β-catenin-Tcf/Lef signalling in both, intestinal crypts and mammary gland stem cells [164, 165].

#### Other modes of control

Unlike the above cases, caveolin-1-mediated inhibition of iNOS involves enhanced degradation of this protein *via* the proteasome pathway. Caveolin-1 recruits iNOS to detergent-insoluble microdomains, presumably through an interaction that requires the segment 101–135 of caveolin-1, a region that may harbour a putative WW-like domain. Components of the proteasome complex were also found in these microdomains [51, 52].

Alternative modes of action involve phosphorylation of caveolin-1 on Tyr14 by src-family kinases. Subsequent recruitment of cytosolic COOH-terminal src kinase (Csk) to caveolin-1 then promotes inactivation of src kinases [166, 167]. Phosphorylation of caveolin-1 on Tyr-14 is observed in response to insulin stimulation [36–38], as well as in response to extracellular stimuli, such as ultraviolet irradiation, H_2_O_2_ and hyperosmolarity. Thus, phosphorylation of caveolin-1 at this site may represent an important element in cellular stress responses [46, 167]. Interestingly, phosphorylation of caveolin-1 on Tyr-14 promotes anchorage-independent growth and cell migration *via* a Grb7-dependent mechanism [39]. Furthermore, src-dependent phosphorylation of caveolin-1 augments its association with type-I matrix metallopro-teinase [168]. Alternatively, however, phosphorylation at this site is considered to be a crucial event in integrin-regulated membrane microdomain internalization [84, 169], as well as EGF-induced caveolae formation [40].

### Cell proliferation

Earlier work by Lisanti and collaborators showed that caveolin-1 modulated the expression of cyclin D1 *via* a transcriptional mechanism involving the Wnt-β-catenin-Tcf/Lef [144, 155]. Subsequent studies in fibroblasts indicated that expression of caveolin-1 inhibited cell proliferation by arresting cells in the G_0_/G_1_ phase of the cell cycle and decreasing the S phase population. These changes were mediated by a mechanism dependent on p53/p21^WAF1/CIP1^[145]. Moreover, expression of caveolin-1 in primary fibroblast cultures promoted a senescent cell phenotype *via* p53/p21 and, conversely, induction of senescence in cells increased endogenous caveolin-1 levels [146]. Interestingly, combined loss of caveolin-1 and the tumour suppressor INK4α in mouse embryonic fibroblasts led to enhanced proliferation *via* changes in the cell cycle that are accompanied by increased cyclin D1 and PCNA levels, down-regulation of p21 ^WAF1/CIP1^ and enhanced p42/44 MAP kinase signalling [170].

Although initial data restricted caveolin-1-mediated control of the cell cycle to the G_0_/G_1_ and S phases, more recent evidence indicates that caveolin-1 expression also reduces the number of cells in G_2_/M while augmenting the sub G_0_/G_1_, apoptotic population. These alterations were associated with changes in survivin, rather than cyclin D1 expression [143]. In support of the notion that caveolin-1 modulates passage through the G_2_/M checkpoint, the chemotherapeutic drug, taxol, was shown to induce caveolin-1 accumulation and G_2_/M arrest in A549 lung cancer cells before these cells commit to cell death [171].

In summary, both inhibition of cyclin D1 and survivin expression reduce cell proliferation. The extent to which one or the other of these two mechanisms is operational downstream of caveolin-1 appears to depend on the cellular context [143]. This conclusion is further sustained by the fact that caveolin-1 presence is also associated with enhanced cell cycle progression in hormonedependent breast cancer cells stimulated with a progesterone analogue [42]. The precise mechanisms that explain such differential responsiveness to caveolin-1 presence remain to be defined (see Fig. 1).

### Cell death and apoptosis

Although a considerable body of evidence supports the notion that caveolin-1 sensitizes, predisposes or directly promotes cell death (mainly apoptosis), some reports suggest an opposite situation. Initial studies showed that caveolin-1 expression sensitizes NIH3T3 fibroblasts and T24 bladder carcinoma cells to staurosporine-induced apoptosis, as assessed by DNA fragmentation, focal adhesion kinase cleavage and changes in nuclear and plasma membrane morphology [119]. In a correlative study, treatment of macrophages with several pro-apoptotic agents or glucose deprivation promoted cell death and increased caveolin-1 at the cell surface, where it colocalized with annexin [172]. In vascular smooth muscle cells, the presence of caveolin-1 ‘switched’ the effect of PDGF from a prolif-erative to a pro-apoptotic signal by down-regulating cyclin D1 and increasing caspase-9 cleavage [173].

Intriguingly, two independent studies showed that caveolin-1 sensitizes L929 fibrosarcoma, HEK293T and HeLa cells to different cytotoxic insults, such as TNFa, H2O2, staurosporine and arsenite *via* activation of the PI3K/Akt pathway [174, 175]. Caveolin-1-mediated activation of Akt was associated with reduced ceramide synthesis [175]. This observation is controversial, given that caveolin-1 reportedly promotes ceramide-mediated cell death *via* inhibition of the PI3K/Akt pathway [176]. Furthermore, cells lacking endogenous caveolin-1 are resistant to apoptosis and, in this case, inhibition of the PI3K/Akt pathway promotes apoptosis [119].

Although apoptosis appears to be the predominant mode of cell death promoted by caveolin-1 presence, the mechanisms underlying these events are still not well defined. Caveolin-1 has been shown to promote caspase-3 activation [119] and data from our laboratory implicated caspase-9, but not caspase-8, as assessed by caspase-9 cleavage and selective inhibition of apoptosis using z-LEHD-fmk (Torres and Quest, unpublished data). Also caveolin-1 tyrosine phosphorylation was recently shown to sensitize MCF7 breast cancer cells to paclitaxel-induced apoptosis by inactivating Bcl2 and increasing mitochondrial permeability [177]. Taken together, these results suggest that caveolin-1 promotes activation of the intrinsic cell death pathway. Indeed, cell cycle changes and apoptosis induced by caveolin-1 expression are prevented by ectopic expression of survivin [143] as might be expected given that survivin inhibits the intrinsic apoptosis pathway and ‘default’ apoptosis at the G_2_/M checkpoint of the cell cycle [178].

## Caveolin-1 in cancer

Cancer is a multi-factorial process that involves loss of a cell's ability to respond to cues provided by the microenvironment in an appropriate fashion. During tumorigenesis, different mechanisms contribute to development of ‘acquired capacities' that explain such autonomous behaviour [179]. The underlying molecular changes are brought about by both genetic mutations and epigenetic mechanisms. According to whether changes involve a ‘gain of function’ or a ‘loss of function’, the molecules are classified as either oncogenes or tumour suppressors. Interestingly, however, caveolin-1 appears to belong to a very select group of proteins that displays both characteristics. Whether caveolin-1 acts to prevent or promote tumour development appears to depend on the cellular context. In the following paragraphs, evidence indicating that caveolin-1 displays traits consistent with a role as a tumour suppressor and/or tumour promotor is summarized.

### The tumour suppressor hypothesis

In the past 10–15 years, a large amount of data have accumulated associating changes in caveolin with the process of cell transformation and indicating that caveolin-1 functions as a tumour suppressor. Caveolin-1 was first described as a highly tyrosine phosphorylated substrate in Rous sarcoma virus-transformed fibroblasts suggesting a role for the protein in the transformation process [34, 35]. Later on, caveolin-1 mRNA and protein levels were shown to be down-regulated in oncogene-transformed fibroblasts in culture [141] and re-expression of caveolin-1 was sufficient to revert the transformed phenotype and prevent anchorage independent growth of these cells [142]. In addition, caveolin-1 down-regulation using antisense oligos is sufficient to drive NIH3T3 cell transformation [117]. These results indicate that loss of caveolin-1 is necessary and sufficient to promote cell transformation, at least in some cell lines, supporting the notion that it functions as a tumour suppressor [18, 60]. Indeed, caveolin-1 expression is reduced in several human tumours, including lung [66, 180, 181], mammary [182], colon [50, 163] and ovarian carcinomas [183] and sarcomas [184], as well as osteosarcomas [185] and re-expression of caveolin-1 often, but not always (see [121, 186–190] and following section) results in reversal of characteristics associated with the transformed phenotype. Consistent with these results, lung hyperplasia, predisposition to mammary as well as carcinogen-induced skin hyperplasia or tumour formation and enhanced angiogenesis are observed in caveolin-1 knockout mice [9, 10, 118, 191–193]. In view of the fact that elimination of caveolin-1 expression *per se* does not increase the spontaneous incidence rate of tumour formation *in vivo* in knockout mice, but rather promotes susceptibility to tumour formation in response to additional insult(s), it may be more appropriate to consider caveolin-1 as a ‘tumour susceptibility’ rather than a tumour suppressor gene.

Also, evidence to the contrary is available. For instance, absence of caveolin-1 expression in primary mammary tumours and tumour-derived cell lines was linked to absence of the protein in primary ductal epithelial cells rather than suppression of expression *via* promotor methylation [194]. Additionally, hematopoietic cells that normally do not express caveolin-1 have been shown to augment caveolin-1 levels in certain states of cell activation and it has been suggested that the caveolin-1 expression may serve as a useful marker for the diagnosis of advanced T-cell leukemia [195]. Related aspects are discussed in the following section.

### Caveolin-1 in multi-drug resistance and metastasis

Despite the extensive list of evidence favouring the role of caveolin-1 as a tumour suppressor, data are also available in the literature supporting an alternative, even opposite view of caveolin-1 as a protein that promotes more aggressive traits in tumour cells, such as metastasis and multi-drug resistance. In normal prostate tissue caveolin-1 is absent, but expression increases upon tumour formation in mouse models and human patients [121] and caveolin-1 presence promotes tumour growth [186] and metastasis of prostate cancer cells *via* an autocrine/paracrine mechanism [188, 189]. In patients, caveolin-1 presence is associated with angiogenesis, aggressive cancer recurrence, elevated metastasis of prostate tumours and poorer patient prognosis [121, 187, 190].

One might argue that such aberrant behaviour of caveolin-1 in the case of prostate (and T cell leukaemia cell lines, see [195]) could be linked to the fact that the protein is not expressed in normal tissue. However, a wealth of data is accumulating indicating that even in tissues where tumour formation is associated with an initial loss of caveolin-1, re-expression of the protein at later stages of tumour development correlates with more malignant tumour characteristics (see Fig. 2). Specifically, in colon cancer cells selected for resistance to methotrexate (HT29-5M21 or M12) or with higher metastatic potential (Lovo E2 versus LovoE5), caveolin-1 levels are elevated with respect to those detected in the wild-type cells [163]. Furthermore, HT29(US) cells, which were obtained from secondary metastasis after injection into nude mice of primary tumour-derived HT29 cells, showed increased endogenous caveolin-1 when compared to the parental HT29 cells (Bender and Quest, unpublished data). Likewise, caveolin-1 expression is increased in multi-drug resistant MCF7 breast cancer cells [196, 197] and caveolin-1 expression in these cells promotes anchorage-independent survival by preventing anoikis [198, 199]. In breast carcinomas, caveolin-1 overexpression has also been associated with metastasis and poor patient prognosis [200]. Likewise, others detected caveolin-1 in myoepithelial cells, endothelial cells and a subset of fibroblasts, but not in luminal epithelial cells (see also [194]). Rather caveolin-1 staining was readily detected in metaplastic breast cancers and to a lesser extent in invasive breast cancers. In the latter case, caveolin-1 expression was associated with reduced patient survival. These results questioned the role of caveolin-1 as a tumour suppressor in basal-like breast carcinomas [201]. Similarly, in patients with non-small cell lung cancer and esophageal squamous cell carcinoma increased expression of caveolin-1 correlates with poor patient prognosis [202, 203].

Furthermore, reduction in caveolin-1 expression was observed at the mRNA and protein level for neoplastic mucosa samples from patients with gastric cancer and caveolin-1 expression was low in cell lines derived from primary tumours. Interestingly, however, caveolin-1 expression increased in cell lines derived from distant metastases, suggesting that stage-dependent fluctuations in caveolin-1 expression (initial suppression followed by re-expression at later stages, see Fig. 2) may contribute to the pathogenesis of gastric cancer [204].

Interestingly, caveolin-1 is associated with polarized distribution of cell signalling components and caveolin-1 is required for cell polarization and migration in two and three dimensions [148, 205, 206]. Consistent with a role for caveolin-1 in migration, re-expression of caveolin-1 in lung adenocarcinoma cells is sufficient to promote filopodia formation, cell migration and metastatic potential of these cells [66]. Furthermore, phosphorylation of caveolin-1 on tyrosine favours cell migration and anchorage-independent growth *via* the adaptor protein Grb7 [39]. Also, caveolin-1 favours directional migration and cell polarization *via* intermediate filament binding [207], as well as a mechanism involving src kinase and Rho GTPases [208]. Alternatively, however, overexpression of caveolin-1 in osteosar-comas abolishes the metastatic ability of these cells by reducing activity of c-src and c-met [185].

In summary, some data indicate that caveolin-1 functions as a tumour suppressor; however, there are equally convincing data associating the presence of caveolin-1 with more aggressive tumour phenotypes even in cells derived from tumours where loss of caveolin-1 is thought to represent an early event in the transition towards a tumour cell (Fig. 2). A possible explanation for these discrepancies is that caveolin-1 functions as a tumour suppressor in systems where negative signalling events downstream of caveolin-1 prevail (Fig. 1A). Alternatively, positive caveolin-1-mediated signalling is likely to be more important in those cases where presence of the protein is associated with more aggressive tumour behaviour (Fig. 1B). Perhaps, one way to resolve the above dilemma is by defining caveolin-1 as a ‘conditional tumour suppressor’ protein.

In this respect, the recent discovery that caveolin-1 requires presence of E-cadherin in cells to display characteristics associated with its role as a tumour suppressor, is of considerable interest [156]. Specifically, caveolin-1 was shown to inhibit β-catenin-Tcf/Lef-dependent transcription of genes associated with cell transformation, like survivin, only when E-cadherin is expressed. Therefore, it is tempting to propose as a working model that caveolin-1 effects in a given cancer cell depends on the ‘transformation status’. In primary cancer cells, where E-cadherin is often still expressed, caveolin-1 exerts its anti-proliferative and pro-apoptotic properties, but in highly metastatic cells from colon or melanoma, this ability is lost [156]. Although we still do not know to what extent the absence of E-cadherin modulates other functions of caveolin-1, loss of E-cadherin is frequently associated with metastasis of tumour cells and thus represents an interesting candidate protein to begin to understand how alterations in cellular ‘context’ contribute to this dramatic switch in caveolin-1 function.

## Concluding remarks

From the onset, caveolin-1 was ascribed a role in processes leading to cell transformation. Despite the plethora of pathways that have been identified since then linking caveolin-1 function to cancer, the precise role of this protein remains unclear. Specifically, caveolin-1 triggers signalling events that are consistent with a role both as a tumour suppressor or as a tumour promotor (Fig. 1). Thus, while it is clear that caveolin-1 function in tumour cells depends on the cellular context, the precise molecular determinants that define how one set of characteristics prevails over the other remain essentially undefined. A crucial issue that needs to be addressed is to define the cellular changes that occur during tumour progression responsible for converting a restrictive cell environment in which caveolin-1 functions as a tumour suppressor to a permissive cell environment where caveolin-1 presence is associated with malignant tumour cell behaviour (Fig. 2). In this respect, the recent discovery that E-cadherin is required for caveolin-1 to inhibit β-catenin/Tcf-Lef-dependent transcription of the inhibitor of apoptosis protein survivin is likely to represent a helpful advance in the field.
